# Assessing Antiangiogenic Therapy Response by DCE-MRI: Development of a Physiology Driven Multi-Compartment Model Using Population Pharmacometrics

**DOI:** 10.1371/journal.pone.0026366

**Published:** 2011-10-18

**Authors:** Andreas Steingoetter, Dieter Menne, Rickmer F. Braren

**Affiliations:** 1 Division of Gastroenterology and Hepatology, University Hospital Zurich, Zurich, Switzerland; 2 Institute for Biomedical Engineering, University and ETH Zurich, Zurich, Switzerland; 3 Menne Biomed Consulting, Tübingen, Germany; 4 Institute of Radiology, Klinikum rechts der Isar der Technischen Universität München, Munich, Germany; National Cancer Institute, United States of America

## Abstract

Dynamic contrast enhanced (DCE-) MRI is commonly applied for the monitoring of antiangiogenic therapy in oncology. Established pharmacokinetic (PK) analysis methods of DCE-MRI data do not sufficiently reflect the complex anatomical and physiological constituents of the analyzed tissue. Hence, accepted endpoints such as Ktrans reflect an unknown multitude of local and global physiological effects often rendering an understanding of specific local drug effects impossible. In this work a novel multi-compartment PK model is presented, which for the first time allows the separation of local and systemic physiological effects. DCE-MRI data sets from multiple, simultaneously acquired tissues, i.e. spinal muscle, liver and tumor tissue, of hepatocellular carcinoma (HCC) bearing rats were applied for model development. The full Markov chain Monte Carlo (MCMC) Bayesian analysis method was applied for model parameter estimation and model selection was based on histological and anatomical considerations and numerical criteria. A population PK model (MTL3 model) consisting of 3 measured and 6 latent (unobserved) compartments was selected based on Bayesian chain plots, conditional weighted residuals, objective function values, standard errors of model parameters and the deviance information criterion. Covariate model building, which was based on the histology of tumor tissue, demonstrated that the MTL3 model was able to identify and separate tumor specific, i.e. local, and systemic, i.e. global, effects in the DCE-MRI data. The findings confirm the feasibility to develop physiology driven multi-compartment PK models from DCE-MRI data. The presented MTL3 model allowed the separation of a local, tumor specific therapy effect and thus has the potential for identification and specification of effectors of vascular and tissue physiology in antiangiogenic therapy monitoring.

## Introduction

Pharmacokinetic (PK) analysis of dynamic contrast enhanced (DCE-) MRI data is widely applied in oncology for the measurement of vascular and tissue physiology. Extracted parameters are used for the characterization and classification of disease processes and for the monitoring of treatment effects. Established PK models are limited to one or two compartments models, considering only the tumor and local plasma compartment [1], [2], and rely on the knowledge of the contrast agent (CA) concentration time curve in the plasma, i.e. the arterial input function (AIF), to reliably compute the tumor tissue specific model parameters such as the plasma-tissue transfer constants *K* ^trans^ and *k*_ep_ and relative plasma *f_p_* (V_plasma_/V_total_) and interstitial *f_i_* (V_interstitial_/V_total_) distribution volumes. An alternative approach using a CA concentration time curve from a simultaneously acquired reference tissue, such as muscle, circumvents the requirement for an AIF [3]. Although this approach has been demonstrated to account for possible changes in cardiovascular physiology it still requires prior knowledge of *f*_i_ in the reference tissue [4].

In pharmacokinetics, any effective space or physiological mechanism, e.g. vascular resistance, which has a distinct effect on CA distribution, should be included as a compartment in the structural model. It is therefore well known that the above described compartment models do not sufficiently reflect the complex anatomical and physiological constituents of the analyzed tissue and that extracted model parameters are biased by a multitude of unknown physiological effects. Despite these limitations and the lack of unified acquisition and analysis methods, the computed model parameters commonly serve as biomarkers for go and no-go decisions in pharmacology and clinical case management.

The danger of model overestimation, strong parameter correlations, sensitivity to the choice of initial values and numerical instability has prevented the development of more complex multi-compartment models. However, many of these obstacles in model development can be overcome by the employment of rich data sets in combination with population PK analysis. Population PK analysis involves the stochastic evaluation of model parameters including their inter-individual, inter-occasional and random variability, thus assuring robust model development.

In this work a novel multi-compartment population PK model is presented, which for the first time allows the separation of local and global physiological effects. This is demonstrated by the improved model quality after the application of covariables defined by the study protocol that reflect either local tissue specific changes in physiology, i.e. different degrees of tumor necrosis, or global systemic changes in cardiovascular physiology, i.e. differences in anesthesia protocol. Model development and evaluation was based on DCE-MRI data consisting of simultaneously acquired, densely sampled gadopentetate dimeglumine (Gd-DTPA) concentration time curves from muscle, liver and tumor tissue of hepatocellular carcinoma (HCC) bearing rats. Population nonlinear mixed effects modeling was performed using the NONMEM® 7.1 program (ICON, Dublin, Ireland) and model selection was based on physiological and histological considerations and standard numerical criteria.

## Materials and Methods

**Figure 1:**
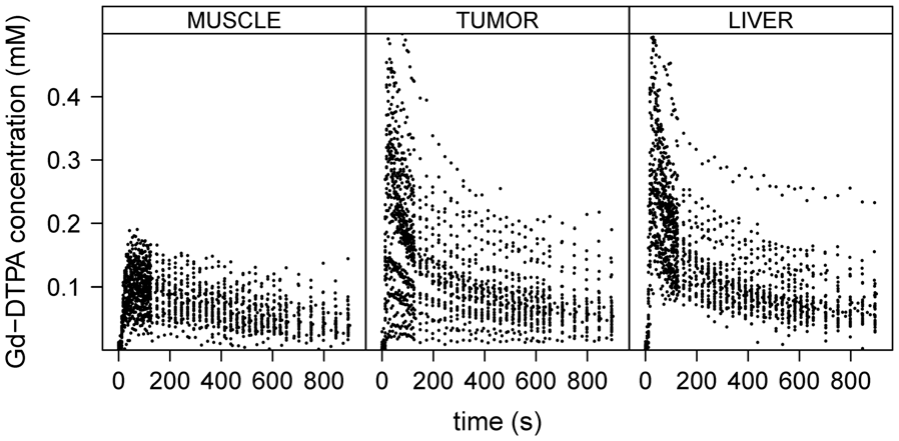
Measured Gd-DTPA concentration data. The measured Gd-DTPA concentration time curves in mM over seconds for the 33 DCE-MRI data sets for spinal muscle tissue (left), tumor tissue (middle) and liver tissue (right). One curve represents the concentration data over time detected for one tissue ROI (muscle, tumor or liver) of one animal at one measurement day. The data show high inter-individual and high inter-occasional variation.

All data were obtained from animal experiments approved by the local ethics committee, Tierschutzkommission der Regierung von Oberbayern (approval ID: 10-06). DCE-MRI data sets with an i.v. bolus injection of Gd-DTPA (0.2 mmol Gd/kg, Magnevist®, Bayer Schering, Germany, Berlin) were acquired in 20 male buffalo rats with implanted unifocal HCC. Animals were measured before and 3 days after transarterial embolization. Before treatment, all animals were anesthetized by injection anesthesia using a mixture of midazolam, medetomidine and fentanyl. After treatment, a subgroup of animals was anesthetized by gaseous infusion of isoflurane. Finally, animals were sacrificed for tumor histology [4]. A total of 33 DCE-MRI data sets were included for model building and selection. Each data set consisted of Gd-DTPA concentration curves derived from defined regions of interest (ROI) in spinal muscle, tumor and liver tissue. Spinal muscle data was chosen as a reference tissue [3], which is always present in abdominal MRI images and was considered unaffected by the applied (tumor specific) treatment, however, influenced by the systemic changes induced by the anesthesia. Gd-DTPA concentration curves consisted of 150 data points with 6 s and 24 s temporal resolutions until 3 min and 15 min, respectively. The defined tumor ROIs covered the central part of the tumors regardless of present necrosis. The acquired Gd-DTPA concentration time curves are shown in **Figure 1**. Based on the histological analysis, treated tumors were classified into three groups with different percentage of residual vital tissue (*vti*) in tumor, i.e. 93–100% (n = 5), 50–93% (n = 6) and below 50% (n = 5). Tumors before treatment were included as a fourth group without histological data (n = 17).

### Structural model selection and evaluation

All population PK analyses were performed by means of the full Markov-Chain Monte-Carlo (MCMC) Bayesian analysis method using NONMEM® 7.1. A random lag term was computed for each record to compensate for variations in the injection time point and treated as a nuisance parameter uncorrelated to the PK model parameters. A mixed proportional and additive error model was used. Prior to the Bayesian analysis, initial parameter estimates were obtained from 120 iterations of the stochastic approximation expectation maximization (*SAEM*) method. After a burn-in phase of 3000 samples in the Bayesian analysis, another 3000 samples were used for parameter estimation. Model building and selection was based on histological (tumor necrosis) and anatomical (i.e. tumor location within the liver) considerations and on numerical criteria, i.e. stability of resulting Bayesian chain plots (*CPS*), conditional weighted residuals (*CWRES*), objective function value (*OFV*), standard error (*SE*) and 95% confidence interval (*95%-CI*) of model parameters in that order as listed. In case of comparable numerical model quality, the deviance information criterion (*DIC*) was computed to allow for Bayesian model comparison [5]. DIC was calculated as 
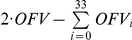
 where the *OFV_i_* are the per-dataset *OFV* values from NONMEM's phi-file.

**Figure 2:**
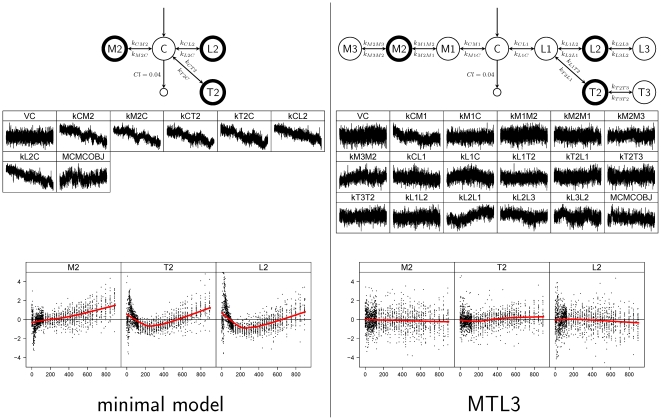
Comparison between the minimal model and the developed MTL3 model. On the left, the schematic of the 4-compartement minimal model including the transfer constants is presented in the top panel consisting of the measured spinal muscle (M2), tumor (T2) and liver (L2) tissue and the latent (not measured) central compartment (C). In the middle and bottom panel, respective *CPS* and *CWRES* of this model are displayed. On the right, the schematic of the 9-compartment MTL3 model including the transfer constants is presented in the top panel consisting of the 3 measured compartments (M2, T2, L2) pointed out by the thick border and the 6 latent (unobserved) compartments (C, M1,M3, T3, L1, L3). In the middle and bottom panel, respective *CPS* and *CWRES* of this model are displayed. All *CPS* plots display the 3000 samples used for Bayesian parameter estimation. The extracted population estimates and 95% confidence intervals for the MTL3 model are provided in Table 1.

The 4-compartment minimal model consisted of the three measured tissue compartments branching off the unobserved (latent) central compartment. The latent central compartment is required in the model as the dose compartment (Gd-DTPA injection) and as the mathematical and physiological interacting compartment mediating the interaction between the measured tissue compartments. As displayed in **Figure 2** **(left)**, it exhibited unsteady *CPS* and systematic trends in *CWRES* indicating the need for additional latent compartments. This model instability was invariable even after applying up to 10000 samples for the Bayesian model estimation. Extending this minimal model by adding serial latent compartments to spinal muscle, tumor and liver, a numerically stable model with minimal systematic trend in *CPS* and *CWRES* was developed and is shown in **Figure 2** **(right)**. This selected final structural model, now called MTL3 model, consisted of 3 measured and 6 latent (unobserved) compartments. The definition of the inter-compartmental transfer constants in the MTL3 model is provided in **Table 1** and the NONMEM code of the MTL3 model is provided in **Text S1** in the supplements. For all models, the clearance was fixed to *Cl* = 0.04 ml⋅s^−1^ based on reported rat clearance values [6].

**Table 1:** Definition of transfer constants in the MTL3 model.

Transfer constant	Definition		
**Muscle transfer constant**	**Central to peripheral**		
***k*** **_CM1_**	C (central)	**→**	M1 (1^st^ muscle)
***k*** **_M1M2_**	M1 (1^st^ muscle)	**→**	M2 (2^nd^ muscle)
***k*** **_M2M3_**	M2 (2^nd^ muscle)	**→**	M3 (3^rd^ muscle)
	**Peripheral to central**		
***k*** **_M3M2_**	M2 (2^nd^ muscle)	**←**	M3 (3^rd^ muscle)
***k*** **_M2M1_**	M1 (1^st^ muscle)	**←**	M2 (2^nd^ muscle)
***k*** **_M1C_**	C (central)	**←**	M1 (1^st^ muscle)
**Tumor transfer constant**	**Central to peripheral**		
***k*** **_L1T2_**	L1 (1^st^ liver)	**→**	T2 (1^st^ tumor)
***k*** **_T2T3_**	T2 (1^st^ tumor)	**→**	T3 (2^nd^ tumor)
	**Peripheral to central**		
***k*** **_T3T2_**	T2 (1^st^ tumor)	**←**	T3 (2^nd^ tumor)
***k*** **_T2L1_**	L1 (1^st^ liver)	**←**	T2 (1^st^ tumor)
**Liver transfer constant**	**Central to peripheral**		
***k*** **_CL1_**	C (central)	**→**	L1 (1^st^ liver)
***k*** **_L1L2_**	L1 (1^st^ liver)	**→**	L2 (2^nd^ liver)
***k*** **_L2L3_**	L2 (2^nd^ liver)	**→**	L3 (3^rd^ liver)
	**Peripheral to central**		
***k*** **_L3L2_**	L2 (2^nd^ liver)	**←**	L3 (3^rd^ liver)
***k*** **_L2L1_**	L1 (1^st^ liver)	**←**	L2 (2^nd^ liver)
***k*** **_L1C_**	C (central)	**←**	L1 (1^st^ liver)

### Covariate model building

Anesthesia protocol and percentage of residual vital tissue in tumor were included as binary (*anest*) and ordered categorical with 4 levels (*vti*) covariables, respectively, into the MTL3 model. Covariate model building was based on physiological observations and histological considerations supported by the generalized additive model (GAM) procedure [7]. Covariable *anest* was applied to spinal muscle or liver transfer constants and *vti* was only applied to the tumor transfer constants, i.e. *k*_T2T3_ or *k*_T3T2_. The MTL3 model was nested within all covariate models and the *OFV*s were compared using the *DIC* criterion.

### Model diagnostics and statistics

All diagnostic plots for model building and evaluation were created using the R software package [8] and the xpose4 library [9]. The *THETA*s, *ETA*s and the respective standard errors *SE*s [%] were computed by NONMEM® 7.1 [10]. The *95%-CI* was computed by the R package coda (http://CRAN.R-project.org/package=coda) [11].

## Results

### Structural model selection and evaluation

The population estimates of the transfer constants with their respective *95%-CI* as well as the *SE*s of the model parameters in the MTL3 model are listed in **Table 2**. The resulting individual concentration curves of spinal muscle, tumor and liver tissue are presented in **Figure S1**, **S2** and **S3**, respectively, in the supplements. The computed correlation matrix is displayed in **Figure S4**.

**Table 2:** *THETA*s of model transfer constants in the MTL3 model with respective *95%-CI* and *SE* of all model parameters.

Transfer constant	Population estimate *THETA* [s^−1^]	Lower Bound [s^−1^]	Upper Bound [s^−1^]	*SE* [%] of *THETA*	*SE* [%] of *ETA*	*SE* [%] of *ERR*(1) *ERR*(2)	
***k*** **_C0_ = ** ***Cl*** **/** ***V_c_***	0.013	0.011	0.016	9	29	4	4.3
***k*** **_CM1_**	0.3	0.25	0.38	8	29	4	4.3
***k*** **_M1C_**	0.012	0.0094	0.015	2	27	4	4.3
***k*** **_M1M2_**	3.6	2.7	5.1	13	28	4	4.3
***k*** **_M2M1_**	300	250	370	1	31	4	4.3
***k*** **_M2M3_**	0.24	0.18	0.3	9	34	4	4.3
***k*** **_M3M2_**	0.0024	0.0018	0.0034	2	32	4	4.3
***k*** **_CL1_**	2.9	2.1	3.7	13	30	4	4.3
***k*** **_L1C_**	0.59	0.44	0.8	29	27	4	4.3
***k*** **_L1T2_**	0.03	0.024	0.038	3	29	4	4.3
***k*** **_T2L1_**	0.38	0.27	0.52	17	29	4	4.3
***k*** **_T2T3_**	3.7	2.1	6.1	20	28	4	4.3
***k*** **_T3T2_**	0.33	0.21	0.52	21	28	4	4.3
***k*** **_L1L2_**	0.0045	0.0037	0.0056	2	29	4	4.3
***k*** **_L2L1_**	0.021	0.015	0.031	5	32	4	4.3
***k*** **_L2L3_**	0.027	0.018	0.037	5	35	4	4.3
***k*** **_L3L2_**	2.3e−05	5.2e−06	7.4e−05	6	34	4	4.3

*THETA* – population estimate of transfer constant.

*ETA* – interindividual variability of transfer constant.

*ERR* – additive (1) and proportional (2) residual random error of the MTL3 model.

**Figure 3:**
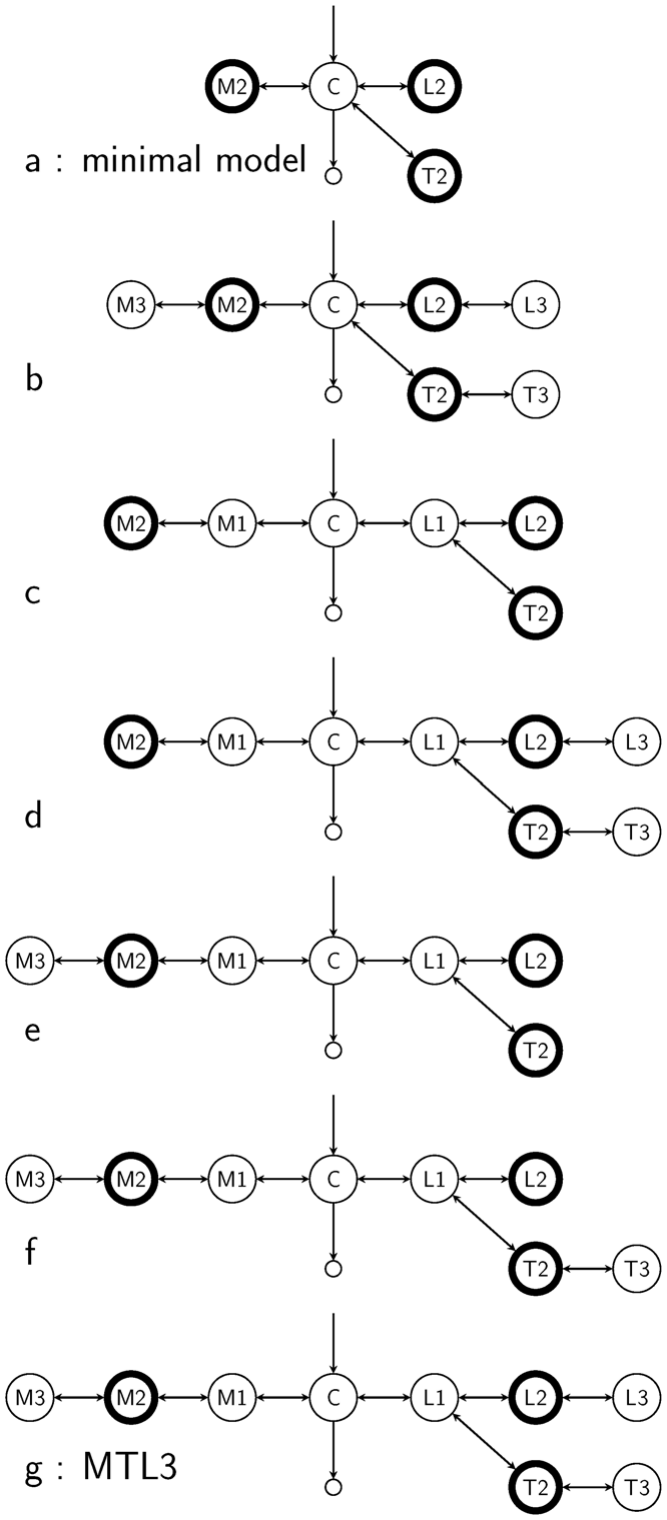
List of model schematics used for the structural model evaluation. Measured compartments are pointed out by the thick border. Models b–f represent the tested reduced models. Model schematic a and g represent the minimal and MTL3 model, respectively.

**Figure 4:**
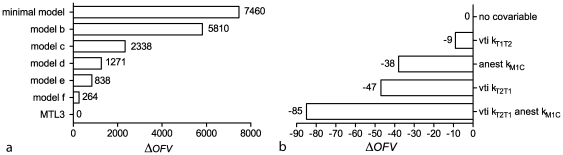
Bar graphs showing the deltas in the *OFV* (Δ*OFV*) relative to the MTL3 model (*OFV* = 0). **a.** Δ*OFV*s for the 4-compartment minimal model and competing reduced models b–f. Based on the deviance information criterion (*DIC*), the MTL3 model exhibited the best fit quality compared to all reduced models. **b.** Δ*OFV* for the covariate models with *SE*s around or below 50% for the included covariates. Based on the deviance information criterion (*DIC*), the covariate models exhibited an improved fit quality compared to the MTL3 model.

The physiologically meaningful or numerically comparable and thus competing reduced models that were used for the structural model evaluation are outlined in **Figure 3**. An overview of these models including the *CPS* and *CWRES* is provided in **Figure S5** in the supplements. The deltas in the objective function value (Δ*OFV*) of the reduced models relative to the full MTL3 model are summarized in the bar graph in **Figure 4a**. The *CPS* and *CWRES* of the reduced models b, c and e show unsteady behavior and systematic deviations and therefore highlight the need for the presence of three spinal muscle compartments in the MTL3 model. The reduced models d and f exhibited the closest numerical outcome compared to the MTL3 model by having similar *CPS* and *CWRES*. For those the deviance information criterion (*DIC*) value was computed to allow a Bayesian model comparison. The resulting *DIC* values of 1130 and 81 supported the superior quality of the MTL3 model.

### Covariate model building

Including the covariables *anest* and *vti* significantly improved the fit quality of the MTL3 model in terms of *OFV* and did not alter the *SE* or *95%-CI* and numerical stability. The maximum drop in the *OFV* value was Δ*OFV* = −85 and achieved by applying both covariables *vti* and *anest* to the tumor transfer constant *k*_T3T2_ and spinal muscle transfer constant *k*_M1C_, respectively. The *THETA*s of the transfer constants with their respective *95%-CI* as well as the *SE*s of the model parameters of this best covariate model are listed in the **Table S1** in the supplements. Model parameter values were comparable to those computed with the MTL3 model without covariable. Covariate models that exhibited *SE*s around or below 50% for the included covariates were considered valid. The Δ*OFVs* of those covariate models are summarized in the bar graph in **Figure 4b** and an overview of the *CPS* and *CWRES* is provided in **Figure S6** in the supplements. The *SE* of the covariate for *vti* was always≤30% and for *anest*≤55%. Applying the covariables to more than two transfer constants simultaneously resulted in model over-parameterization and numerical instabilities. All reliable covariate models indicated an impact of *anest* and *vti* on the observed Gd-DTPA concentration data.

## Discussion

In this work a multi-compartment PK model is developed allowing a more detailed description of Gd-DTPA kinetics. The model requires no use of an arterial input function or fixed tissue parameters. This was achieved by population PK modeling of DCE-MRI data from multiple tissues, i.e. spinal muscle, tumor and liver. Model building and selection were driven by anatomical and histological knowledge and numerical methods commonly applied in pharmacometrics.

The here presented methodological approach for the development, and validation of a multi-compartment PK model for Gd-DTPA underlines the credibility of the here proposed MTL3 model. In fact, the proposed approach is not limited to the three selected tissue ROIs, but can be further extended by observations from additional ROIs or clusters within ROIs. Model development was performed using NONMEM® 7.1, a well recognized and widely applied nonlinear mixed effects modeling tool used for population PK/PD analyses [10]. Population modeling is commonly applied in medical statistics to improve the precision of subject specific treatment effects taking into account inter-individual and inter-occasional variability. The MCMC Bayesian analysis method presents an efficient estimation approach for high dimensional PK problems with rich and correlated data [12]. It has previously been used to develop a Bayesian hierarchical model for MR contrast agent kinetics based on DCE-MRI data [13], [14]. This model proposed by Schmid et al. was based on the simple standard 1-compartment model [2] and required the knowledge and input of an AIF. Their approach has been successfully applied in a phase II oncology study using data-driven parametric AIFs to assess treatment effects [15]. Bayesian methods use prior information, but for the proposed MTL3 model uninformative priors were sufficient to establish convergence (see Text S1). The numerical criteria for model selection applied in this work, i.e. the stability of Bayesian chain plots (*CPS*), conditional weighted residuals (*CWRES*), objective function value (*OFV*) and standard error (*SE*) of model parameters are well accepted plots and measures for basic internal model evaluation [16]. With a total time of 14 hours for a single run without covariables on an Intel Core i7-980X, the MTL3 model allows parameter estimation within an acceptable time range for research applications.

For structural model selection, *CPS* and *CWRES* were the primary selection criteria as these allow a fast visual inspection of quality of fit. From the drift in the *CPS* and the systematic offsets in the *CWRES*, it was obvious that three compartments are required to describe the spinal muscle tissue. The necessity for additional peripheral latent compartments for tumor (T3) and liver (L3) was not as apparent from the residual plots alone. However, the statistical comparison using the deviance information criterion (*DIC*) supported the inclusion of the additional latent compartments. The *DIC* is a widely applied measure for Bayesian model comparison also used in quantitative DCE-MRI [17] and presents a Bayesian analogue of the Akaike information criterion (*AIC*) [5]. These results confirm the feasibility to develop multi-compartment population PK models based on simultaneously acquired DCE-MRI data from multiple tissues.

For covariate model selection, *OFV* and *SE* of model parameters were the primary selection criteria as no differences were detectable by visual inspection of *CPS* and *CWRES*. The selected covariables *vti*, reflecting local tumor necrosis quantified by tumor histological analysis and *anest*, reflecting an induced systemic change in cardiovascular physiology, i.e. 30% change in heart rate detected by ECG recordings represent a local and a systemic factor influencing Gd-DTPA kinetics [4]. Based on these observations and the GAM procedure, the covariable *vt*i was only applied to transfer constants of tumor tissue. Most of the tested covariate models exhibited a *SE* for the included covariable much larger than 50% and thus were considered unreliable, leaving only one *anest* covariate model (*k*_M1C_) and the two *vti* covariate models. When combining the two best covariate models (*vti k*_T3T2_ and *anest k*_M1C_) the fit quality further improved without compromising the *SE* of model parameters. These results confirm an impact of *anest* and *vti* on the measured Gd-DTPA concentration data which is in accordance with previous findings [4]. Furthermore, the successful combined application of both *vti* and *anest* highlight the capability of the MTL3 model to separate local and systemic effects present in Gd-DTPA concentration data.

For all tested models, the clearance was fixed to *Cl* = 0.04 ml s^−1^. The clearance is a mandatory parameter in pharmacokinetic modeling describing the washout of the injected substance from the body. Often in PK modeling, when clearance is not directly measured from blood or urine samples, this parameter is a priori unidentifiable and therefore taken from literature and fixed during model development [18]. We have tested the use of a prior on clearance resulting in longer computation with no improvement in model quality. This indicates that clearance was an ill-defined (unidentifiable) parameter which may rather be determined by direct measurements of Gd-DTPA blood half-lives.

Computed population estimates of model parameters and their respective *95%-CI* showed no difference between the MTL3 and the best covariate model. In both models, there was a large range of values for the computed model transfer constants ranging from 3.5⋅10^−5^ s^−1^ to 3.2⋅10^2^ s^−1^. Using the fixed clearance value of *Cl* = *k*_C0_⋅*V*_c_ = 0.04 ml⋅s^−1^, the MTL3 model's inter-compartmental distributions (*Q*) and the apparent volumes of distribution (*V*) can be calculated from the estimated transfer constants using the relationship *k* = *Q*/*V*. The inter-compartmental distribution can be conceptually viewed as a pharmacokinetic expression of the transport occurring between tissues and organs via blood vessels and/or membranes and was assumed to follow first-order kinetics [19]. The apparent volume of distribution relates the total amount of Gd-DTPA in the body (*A*) to the site of measurement, i.e. spinal muscle, liver or tumor. It can be viewed simply as a proportionality factor between the total amount of Gd-DTPA present in the entire body and the Gd-DTPA concentration *C*_Gd_ in the tissue of interest, i.e. *V* = *A*/*C*_Gd_ [20].

**Figure 5:**
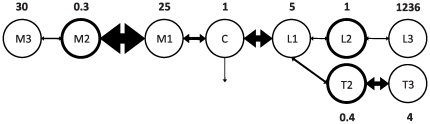
Model schematic of the MTL3 model. The model schematic illustrating the magnitudes of the inter-compartmental distribution (double-headed arrows) between and the normalized apparent distribution volumes (numbers in bold) of the individual compartments (circles) of the MTL3 model. The size of the arrows indicate magnitude of the respective inter-compartmental distribution. Measured compartments are pointed out by the thick border.

A schematic of the MTL3 model illustrating the magnitudes of the inter-compartmental distributions and showing the normalized apparent distribution volumes is presented in **Figure 5**. Very large inter-compartmental transport was determined between M1 and M2, whereas very small inter-compartmental transport was determined between L1 and L2 as well as L2 and L3. High *C*_Gd_ values were detected for M2 and T2, whereas low and very low *C*_Gd_ values were detected for M3, M1 and L3.

At this stage, the MTL3 model must be viewed as a phenomenological population PK model for the in vivo Gd-DTPA distribution kinetics in rats. Based on the results from the covariate model building, the latent tumor compartment T3 may be interpreted as interstitial tissue space, since this is most strongly affected by cellular destruction which is represented by *vti*. Similarly, the latent compartment M1 may be considered a compartment related to either blood vessel space or blood vessel function, e.g. blood flow resistance or blood pressure which is known to be affected by *anest*. The exceptional structural and functional organization of the liver also seems to be reflected in the MTL3 model. The exceptionally large *V*_L3_ resulting in essentially no transport back to compartment L2 (*k*_L3L2_ = 2.3 10^−5^) may reflect the special drainage system via the central hepatic veins. DCE-MRI data of higher spatial resolution and SNR allowing for cluster and pixel based analyses as well as the inclusion of further and/or other reference tissues such as kidney or spleen will extend the model to capture tissue heterogeneity and validate the physiological interpretation of the model.

**Figure 6:**
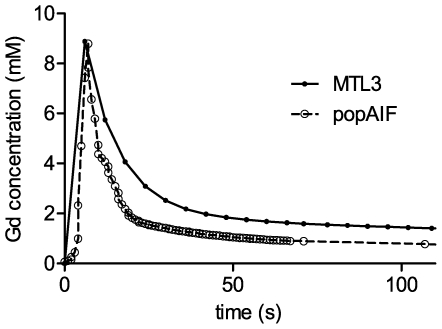
The concentration time curve of central compartment compared with a rat population AIF. The computed Gd-DTPA concentration time curve for the central compartment of the MTL3 model for the analyzed rat population (solid line and dots) and a previously reported rat population AIF derived in the abdominal aorta of genetically similar rats using rapid DCE-CT and a parametric population AIF model (dashed line and open circles) [4].

The MTL3 model does not depend on the knowledge of an arterial input function to calculate the transfer constants for spinal muscle, tumor or liver tissue. Rather, the MTL3 model computes based on the concentration data measured in these peripheral tissues, the Gd-DTPA concentration curve in the central compartment. This may resemble an arterial input function detected in a central vessel like the aorta. **Figure 6** displays the computed Gd-DTPA concentration time curve for the central compartment of the MTL3 model for the analyzed rat population. It is compared to the previously reported rat population AIF [4] measured in the abdominal aorta of genetically similar rats. Visual inspection indicated a close match at peak concentration values, however a slower decrease for the central concentration curve of the MTL3 model.

In conclusion, our multi-compartment MTL3 population PK model demonstrates that tumor compartment covariates can be used as predictors of tumor tissue physiology and that no knowledge of the arterial input function or tissue specific characteristics is required. The presented population PK modeling approach allows a more in-depth analysis of MRI contrast agent distribution kinetics and thus has the potential for enhanced identification and specification of effectors of vascular and tissue physiology. This will be of particular interest in the evaluation of antiangiogenic therapy response in oncology.

## Supporting Information

Text S1 **The NONMEM 7.1 code of the MTL3 model.**
(DOC)

Table S1 ***THETA*** **s of the model transfer constants in the covariate model.** The *THETA*s of the model transfer constants in the covariate model applying *vti* to *k*_T3T2_ and *anest* to *k*_M1C_ with respective *95%-CI* and *SE* of all model parameters.
(DOC)

Figure S1 **Individual measured and fitted Gd-DTPA concentration data for spinal muscle tissue.** The measured (dots), the individual fitted (black line) and the population fitted (grey line) concentration data of each animal before and after treatment are displayed over the entire imaging period of 15.
(TIFF)

Figure S2 **Individual measured and fitted Gd-DTPA concentration data for tumor tissue.** The measured (dots), the individual fitted (black line) and the population fitted (grey line) concentration data of each animal before and after treatment are displayed over the entire imaging period of 15 minutes.
(TIFF)

Figure S3 **Individual measured and fitted Gd-DTPA concentration data for liver tissue.** The measured (dots), the individual fitted (black line) and the population fitted (grey line) concentration data of each animal before and after treatment are displayed over the entire imaging period of 15 minutes.
(TIFF)

Figure S4 **The correlation matrix for the MTL3 model.** The correlation matrix for the MTL3 model parameters as computed by the NONMEM 7.1 program [10]. The correlation coefficient *r* was converted into a flattening factor *f* = 1−*r* which was interpreted as the ratio of the minor to the major axis of an ellipse. The resulting ellipses are displayed and color coded with regard to the value of *f*, i.e. dark blue circle for *f* = 1 and dark red line for *f* = 0.
(TIFF)

Figure S5 **Detailed overview of the tested structural models.** (**a**) The minimal model, (**b–f**) the reduced models and (**g**) the MTL3 model are listed with the respective model schematic (left), *CPS* (middle) and *CWRES* (right). In addition, the computed Δ*OFV*s are shown as number with grey bar (bottom right). Measured compartments are pointed out by the thick border. All *CPS* plots display the 3000 samples used for Bayesian parameter estimation.
(TIFF)

Figure S6 **Detailed overview of the MTL3 model and all valid covariate models.** The *CPS* (left), *CWRES* (right) and Δ*OFV*s (bottom right) of the MTL3 (top), the best covariate model applying *vti* to *k*_T3T2_ and *anest* to *k*_M1C_ (bottom) and the other covariate models that exhibited *SE*s around or below 50% for the included covariates are presented. All *CPS* plots display the 3000 samples used for Bayesian parameter estimation.
(TIF)
