# Supplementary material for: Assessing Antiangiogenic Therapy Response by DCE-MRI: Development of a Physiology Driven Multi-Compartment Model Using Population Pharmacometrics
Source: PLoS One. 2011 Oct 18;6(10):e26366. doi: 10.1371/journal.pone.0026366 (PMC3196562; doi:10.1371/journal.pone.0026366)
Supplement: Text S1 — The NONMEM 7.1 code of the MTL3 model. (DOC) [file pone.0026366.s001.doc]

**Text S1. The NONMEM 7.1 code of the MTL3 model**

$PROB MTL3 Model without covariables. Steingoetter, Menne, Braren 2011

$DATA DataMTL3.txt IGNORE=C

$INPUT ID,TIME,CMT,AMT,DV,MDV,VITTISSUE,VTI,ANEST

$SUBROUTINES ADVAN5

; 1 2 3 4 5 6 7 8 9

$MODEL COMP=C COMP=M2 COMP=T2 COMP=M3 COMP=L2 COMP=L1 COMP=T3 COMP=L3 COMP=M1

$PRIOR NWPRI NTHETA=18, NETA=18, NTHP=0, NETP=18

$PK

MU_1 = THETA(1)

MU_2 = THETA(2)

MU_3 = THETA(3)

MU_4 = THETA(4)

MU_5 = THETA(5)

MU_6 = THETA(6)

MU_7 = THETA(7)

MU_8 = THETA(8)

MU_9 = THETA(9)

MU_10 = THETA(10)

MU_11 = THETA(11)

MU_12 = THETA(12)

MU_13 = THETA(13)

MU_14 = THETA(14)

MU_15 = THETA(15)

MU_16 = THETA(16)

MU_17 = THETA(17)

MU_18 = THETA(18)

; CENTRAL

CL = 0.04

VC = EXP(MU_1+ETA(1))

S2 = VC

S3 = VC

S5 = VC

; CENTRAL

K10 = CL/VC

; MUSCLE

K19 = EXP(MU_2+ETA(2))

K91 = EXP(MU_3+ETA(3))

K92 = EXP(MU_4+ETA(4))

K29 = EXP(MU_5+ETA(5))

K24 = EXP(MU_6+ETA(6))

K42 = EXP(MU_7+ETA(7))

; TUMORLIVER

K16 = EXP(MU_8+ETA(8))

K61 = EXP(MU_9+ETA(9))

; TUMOR

K63 = EXP(MU_10+ETA(10))

K36 = EXP(MU_11+ETA(11))

K37 = EXP(MU_12+ETA(12))

K73 = EXP(MU_13+ETA(13))

; LIVER

K65 = EXP(MU_14+ETA(14))

K56 = EXP(MU_15+ETA(15))

K58 = EXP(MU_16+ETA(16))

K85 = EXP(MU_17+ETA(17))

ALAG1 = EXP(MU_18+ETA(18))

$ERROR

Y=F+F*ERR(2)+ERR(1)

IPRED = F

IRES = DV-IPRED ; INDIVIDUAL-SPECIFIC RESIDUAL

IWRES = IRES ; INDIVIDUAL-SPECIFIC WEIGHTED RESIDUAL

$THETA

1.3 ; VC

-1.4 ; K19

-4.4 ; K91

0.8 ; K92

5.2 ; K29

-1.8 ; K24

-6.3 ; K42

1.0 ; K16

-0.5 ; K61

-3 ; K63

-1.25 ; K36

1.2 ; K37

-0.37 ; K73

-5.2; K65

-5.0 ; K56

-3.2 ; K58

-10. ; K85

2.35 ; ALAG1

$OMEGA BLOCK(17)

0.1 ; VC

0.01 0.1 ; K19

0.01 0.01 0.1 ; K91

0.01 0.01 0.01 0.1 ; K92

0.01 0.01 0.01 0.01 0.1 ; K29

0.01 0.01 0.01 0.01 0.01 0.1 ; K24

0.01 0.01 0.01 0.01 0.01 0.01 0.1 ; K42

0.01 0.01 0.01 0.01 0.01 0.01 0.01 0.1 ; K16

0.01 0.01 0.01 0.01 0.01 0.01 0.01 0.01 0.1 ; K61

0.01 0.01 0.01 0.01 0.01 0.01 0.01 0.01 0.01 0.1 ; K63

0.01 0.01 0.01 0.01 0.01 0.01 0.01 0.01 0.01 0.01 0.1 ; K36

0.01 0.01 0.01 0.01 0.01 0.01 0.01 0.01 0.01 0.01 0.01 0.1 ; K37

0.01 0.01 0.01 0.01 0.01 0.01 0.01 0.01 0.01 0.01 0.01 0.01 0.1 ; K73

0.01 0.01 0.01 0.01 0.01 0.01 0.01 0.01 0.01 0.01 0.01 0.01 0.01 0.1 ; K65

0.01 0.01 0.01 0.01 0.01 0.01 0.01 0.01 0.01 0.01 0.01 0.01 0.01 0.01 0.1 ; K56

0.01 0.01 0.01 0.01 0.01 0.01 0.01 0.01 0.01 0.01 0.01 0.01 0.01 0.01 0.01 0.1 ; K58

0.01 0.01 0.01 0.01 0.01 0.01 0.01 0.01 0.01 0.01 0.01 0.01 0.01 0.01 0.01 0.01 0.1 ; K85

$OMEGA

0.04 ; ALAG1

$SIGMA 5.5E-05 0.0068

$OMEGA BLOCK(17)

0.1 FIX

0 0.1

0 0 0.1

0 0 0 0.1

0 0 0 0 0.1

0 0 0 0 0 0.1

0 0 0 0 0 0 0.1

0 0 0 0 0 0 0 0.1

0 0 0 0 0 0 0 0 0.1

0 0 0 0 0 0 0 0 0 0.1

0 0 0 0 0 0 0 0 0 0 0.1

0 0 0 0 0 0 0 0 0 0 0 0.1

0 0 0 0 0 0 0 0 0 0 0 0 0.1

0 0 0 0 0 0 0 0 0 0 0 0 0 0.1

0 0 0 0 0 0 0 0 0 0 0 0 0 0 0.1

0 0 0 0 0 0 0 0 0 0 0 0 0 0 0 0.1

0 0 0 0 0 0 0 0 0 0 0 0 0 0 0 0 0.1

$OMEGA

0.1 FIX ; ALAG1

$THETA (17.0 FIXED) (1 FIXED)

$EST PRINT=1 METHOD=SAEM INTERACTION NBURN=100 NITER=20 FILE=SAEM.EXT NOPRIOR=1

$EST METHOD=BAYES FILE=BAYES.EXT NBURN = 4000 NSIG = 3 NITER= 3000 NOPRIOR=0

$COV MATRIX=R PRINT=E UNCONDITIONAL SIGL=12
