# Supplementary material for: Assessing Antiangiogenic Therapy Response by DCE-MRI: Development of a Physiology Driven Multi-Compartment Model Using Population Pharmacometrics
Source: PLoS One. 2011 Oct 18;6(10):e26366. doi: 10.1371/journal.pone.0026366 (PMC3196562; doi:10.1371/journal.pone.0026366)
Supplement: Table S1 — THETA s of the model transfer constants in the covariate model. The THETAs of the model transfer constants in the covariate model applying vti to k T3T2 and anest to k M1C with respective 95%-CI and SE of all model parameters. (DOC) [file pone.0026366.s002.doc]

**Table S4. *THETA*s** **of the model transfer constants in the covariate model applying *vti* to *k*_T3T2_ and *anest* to *k*_M1C_ with respective *95%-CI* and *SE* of all model parameters.**

| **Transfer constant** | **Population estimate**  ***THETA* [s^-1^]** | **Lower**  **Bound [s^-1^]** | **Upper**  **Bound [s^-1^]** | ***SE* [%] of**  ***THETA*** | ***SE* [%] of**  ***ETA*** | ***SE* [%] of**  ***ERR*(1) *ERR*(2)** | |
| --- | --- | --- | --- | --- | --- | --- | --- |
| ***k*_C0_ = *Cl*/*V_c_*** | 0.017 | 0.015 | 0.022 | 12 | 37 | 4.1 | 4.4 |
| ***k*_CM1_** | 0.35 | 0.29 | 0.42 | 9 | 28 |
| ***k*_M1C_** | 0.01 | 0.0076 | 0.014 | 3 | 28 |
| ***k*_M1M2_** | 2.9 | 2.3 | 3.6 | 10 | 29 |
| ***k*_M2M1_** | 320 | 260 | 380 | 1 | 29 |
| ***k*_M2M3_** | 0.33 | 0.25 | 0.44 | 13 | 29 |
| ***k*_M3M2_** | 0.0021 | 0.0015 | 0.0029 | 2 | 32 |
| ***k*_CL1_** | 3.7 | 3 | 4.5 | 8 | 29 |
| ***k*_L1C_** | 0.63 | 0.52 | 0.77 | 21 | 38 |
| ***k*_L1T2_** | 0.033 | 0.025 | 0.044 | 4 | 34 |
| ***k*_T2L1_** | 0.56 | 0.44 | 0.73 | 22 | 31 |
| ***k*_T2T3_** | 5.9 | 3.7 | 9.3 | 13 | 27 |
| ***k*_T3T2_** | 0.46 | 0.46 | 0.76 | 33 | 26 |
| ***k*_L1L2_** | 0.0034 | 0.0028 | 0.0041 | 1 | 29 |
| ***k*_L2L1_** | 0.022 | 0.015 | 0.036 | 6 | 30 |
| ***k*_L2L3_** | 0.025 | 0.016 | 0.035 | 5 | 32 |
| ***k*_L3L2_** | 0.000034 | 0.0000075 | 0.00012 | 6 | 45 |
| ***C_anest*** | 1.3 | 0.97 | 1.7 | 54 |  |
| ***C*_*vti*** | 0.73 | 0.6 | 0.88 | 30 |
| *THETA* – population estimate of transfer constant  *ETA* – inter-individual variability of transfer constant  *ERR* – additive (1) and proportional (2) residual random error of the covariate model  *C_anest –* covariate to *anest* applied to *k*_M1C_  *C_vti -* covariate to *vti* applied to *k*_T3T2_ | | | | | | | |
