# Supplementary figures and images for: Assessing Antiangiogenic Therapy Response by DCE-MRI: Development of a Physiology Driven Multi-Compartment Model Using Population Pharmacometrics
Source: PLoS One. 2011 Oct 18;6(10):e26366. doi: 10.1371/journal.pone.0026366 (PMC3196562; doi:10.1371/journal.pone.0026366)

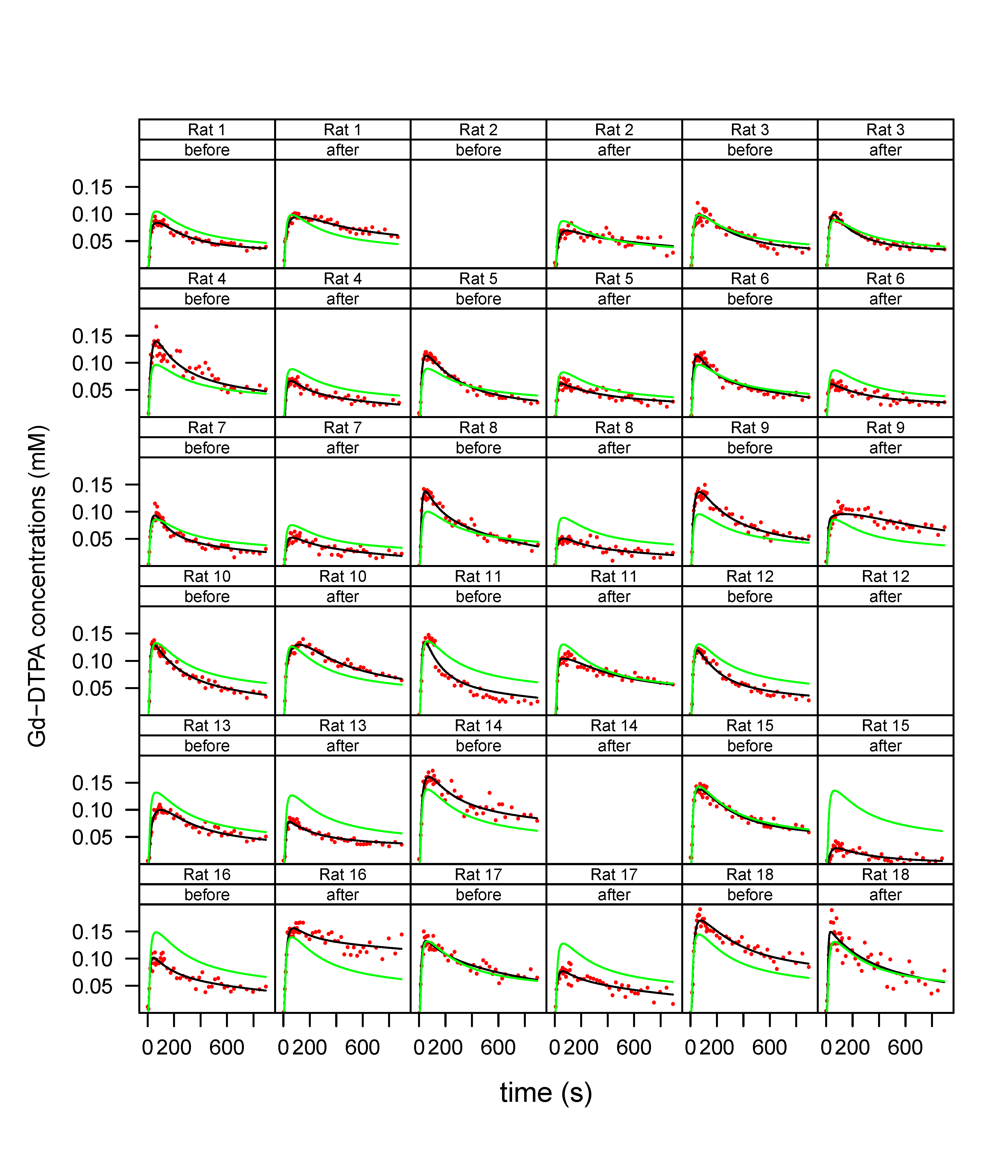

Supplement: Figure S1 — Individual measured and fitted Gd-DTPA concentration data for spinal muscle tissue. The measured (dots), the individual fitted (black line) and the population fitted (grey line) concentration data of each animal before and after treatment are displayed over the entire imaging period of 15. (TIFF) [file pone.0026366.s003.tiff]

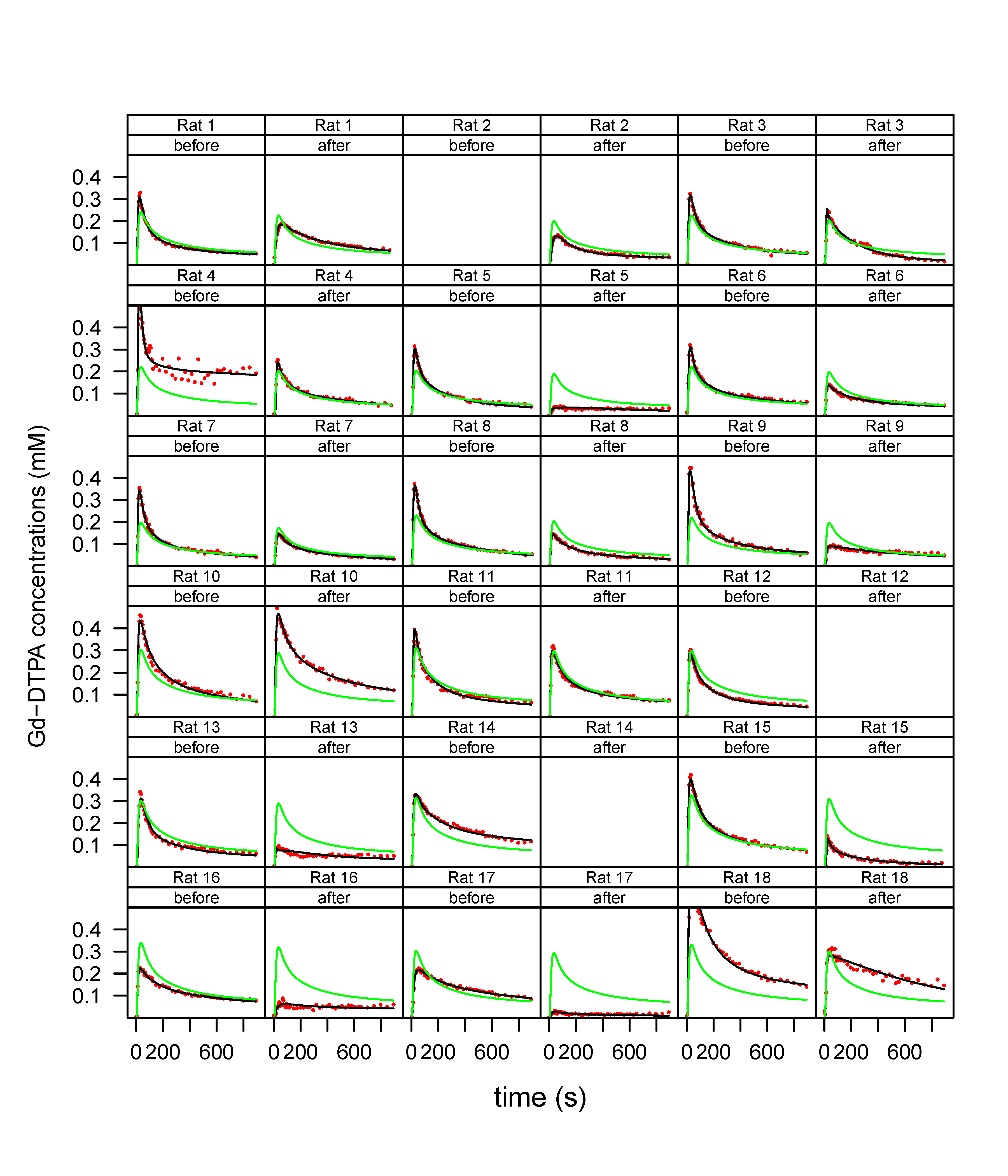

Supplement: Figure S2 — Individual measured and fitted Gd-DTPA concentration data for tumor tissue. The measured (dots), the individual fitted (black line) and the population fitted (grey line) concentration data of each animal before and after treatment are displayed over the entire imaging period of 15 minutes. (TIFF) [file pone.0026366.s004.tiff]

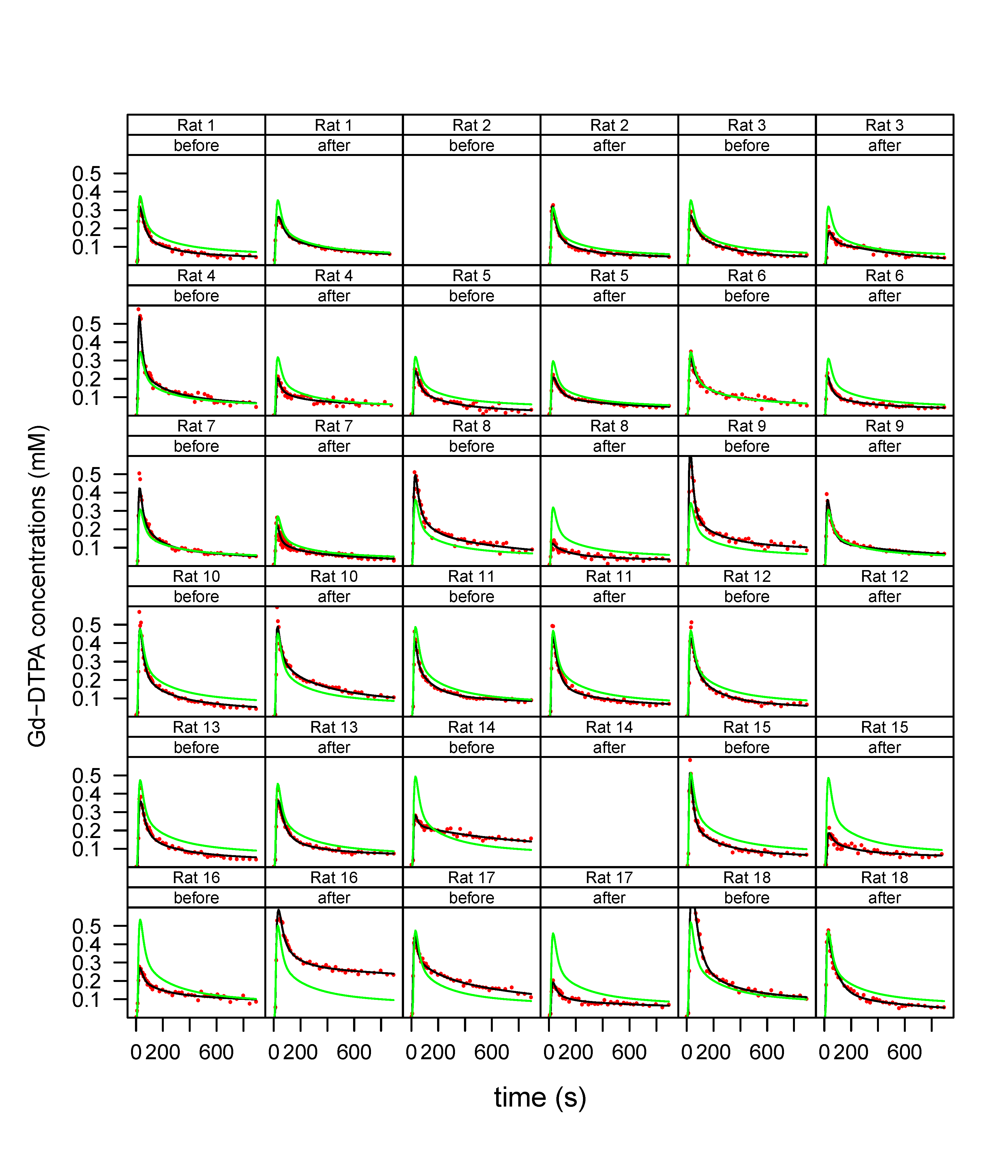

Supplement: Figure S3 — Individual measured and fitted Gd-DTPA concentration data for liver tissue. The measured (dots), the individual fitted (black line) and the population fitted (grey line) concentration data of each animal before and after treatment are displayed over the entire imaging period of 15 minutes. (TIFF) [file pone.0026366.s005.tiff]

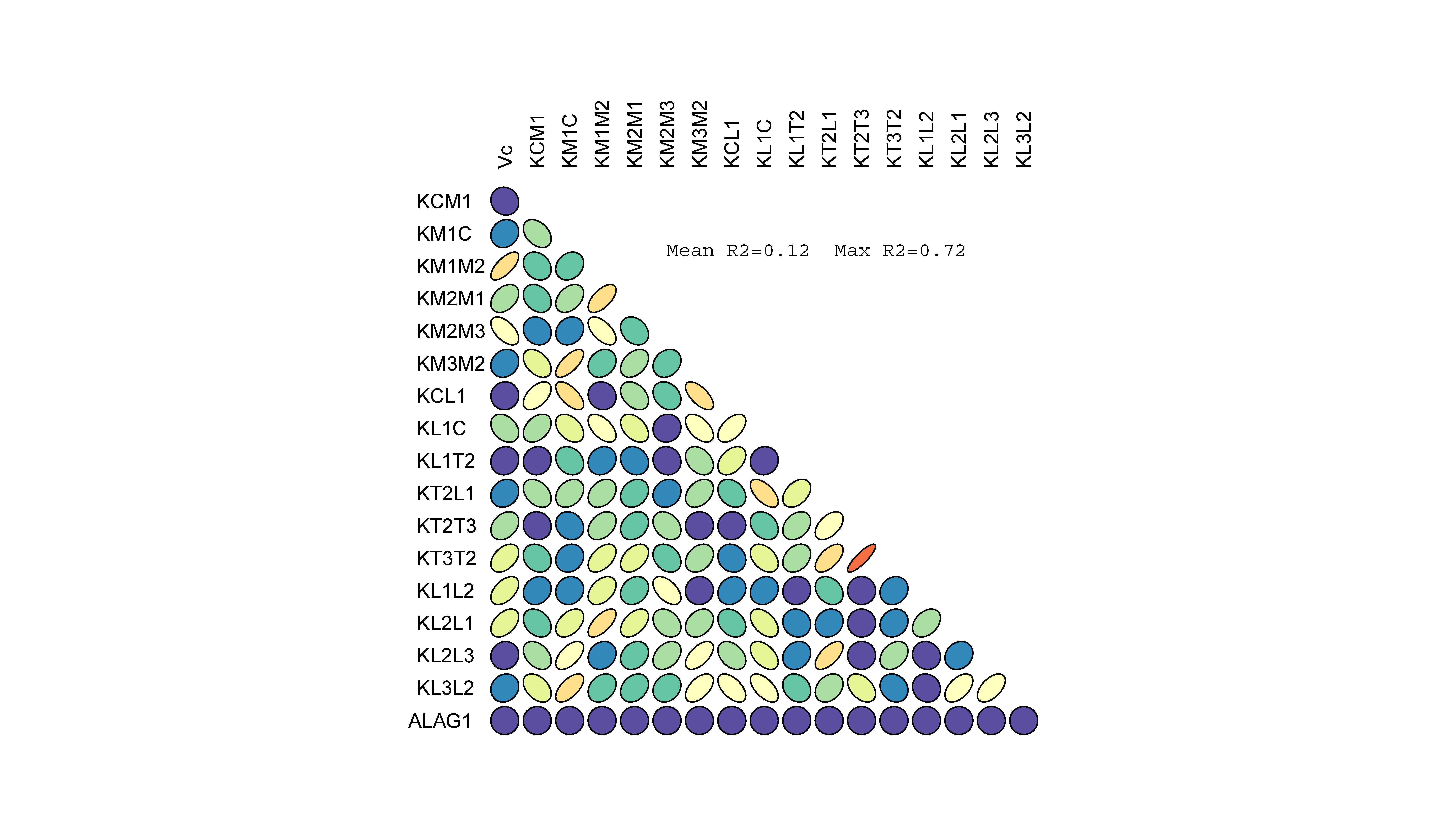

Supplement: Figure S4 — The correlation matrix for the MTL3 model. The correlation matrix for the MTL3 model parameters as computed by the NONMEM 7.1 program [10]. The correlation coefficient r was converted into a flattening factor f = 1−r which was interpreted as the ratio of the minor to the major axis of an ellipse. The resulting ellipses are displayed and color coded with regard to the value of f, i.e. dark blue circle for f = 1 and dark red line for f = 0. (TIFF) [file pone.0026366.s006.tiff]

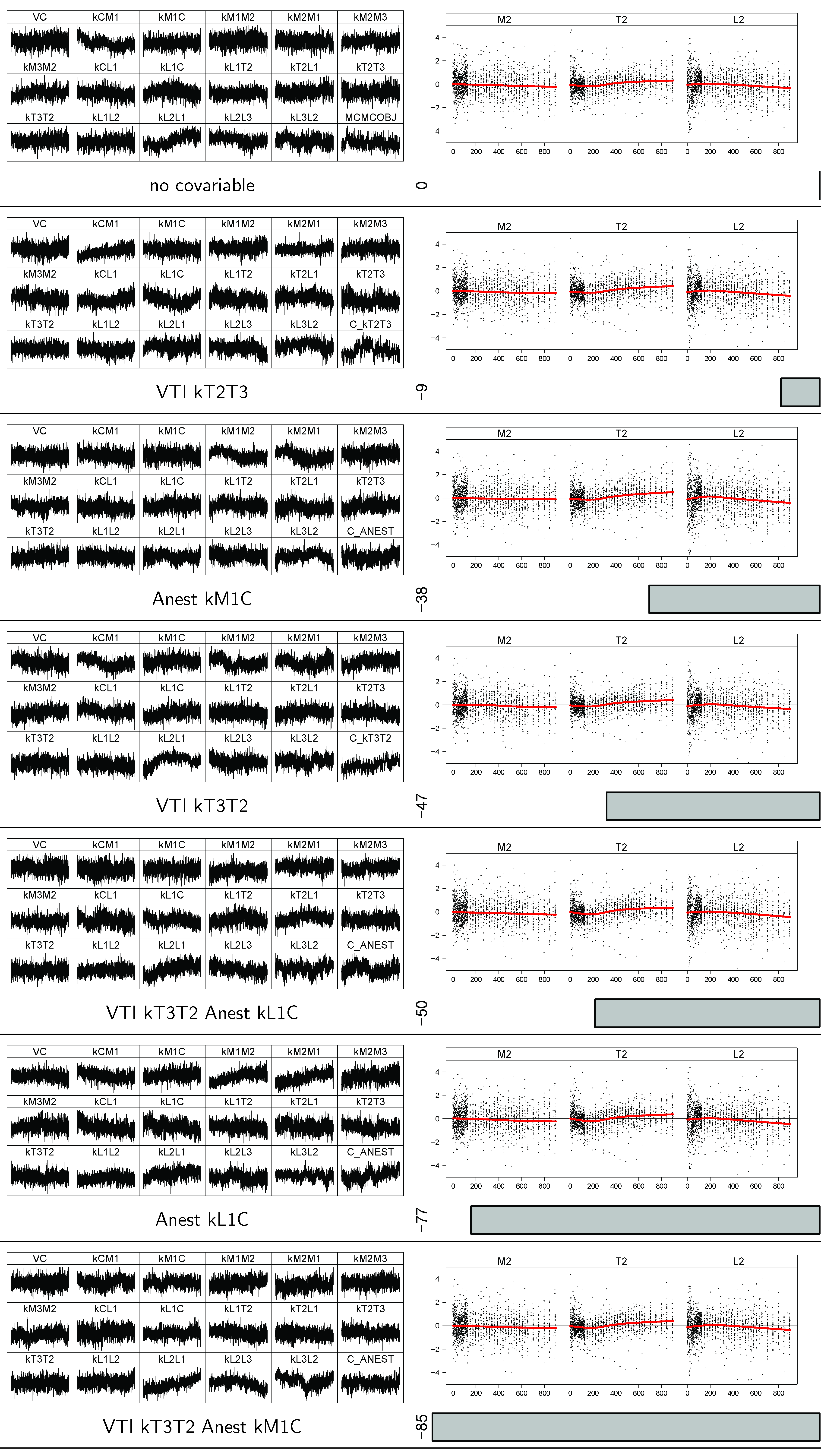

Supplement: Figure S6 — Detailed overview of the MTL3 model and all valid covariate models. The CPS (left), CWRES (right) and ΔOFVs (bottom right) of the MTL3 (top), the best covariate model applying vti to k T3T2 and anest to k M1C (bottom) and the other covariate models that exhibited SEs around or below 50% for the included covariates are presented. All CPS plots display the 3000 samples used for Bayesian parameter estimation. (TIF) [file pone.0026366.s008.tif]
